# Mechanical characterisation of polymer of intrinsic microporosity PIM-1 for hydrogen storage applications

**DOI:** 10.1007/s10853-016-0647-4

**Published:** 2016-12-21

**Authors:** Katarzyna Polak-Kraśna, Robert Dawson, Leighton T. Holyfield, Chris R. Bowen, Andrew D. Burrows, Timothy J. Mays

**Affiliations:** 1 Department of Mechanical Engineering, University of Bath, Claverton Down, Bath, BA2 7AY UK; 2 Department of Chemistry, University of Bath, Claverton Down, Bath, BA2 7AY UK; 3 Department of Chemical Engineering, University of Bath, Claverton Down, Bath, BA2 7AY UK; 4 Doctoral Training Centre in Sustainable Chemical Technologies, University of Bath, Claverton Down, Bath, BA2 7AY UK; 5 Department of Chemistry, University of Sheffield, Brook Hill, Sheffield, S3 7HF UK

**Keywords:** Storage Modulus, Dynamic Mechanical Thermal Analysis, Hydrogen Storage, Failure Strain, Dynamic Mechanical Thermal Analysis

## Abstract

Polymers of intrinsic microporosity (PIMs) are currently attracting interest due to their unusual combination of high surface areas and capability to be processed into free-standing films. However, there has been little published work with regards to their physical and mechanical properties. In this paper, detailed characterisation of PIM-1 was performed by considering its chemical, gas adsorption and mechanical properties. The polymer was cast into films, and characterised in terms of their hydrogen adsorption at −196 °C up to much higher pressures (17 MPa) than previously reported (2 MPa), demonstrating the maximum excess adsorbed capacity of the material and its uptake behaviour in higher pressure regimes. The measured tensile strength of the polymer film was 31 MPa with a Young’s modulus of 1.26 GPa, whereas the average storage modulus exceeded 960 MPa. The failure strain of the material was 4.4%. It was found that the film is thermally stable at low temperatures, down to −150 °C, and decomposition of the material occurs at 350 °C. These results suggest that PIM-1 has sufficient elasticity to withstand the elastic deformations occurring within state-of-the-art high-pressure hydrogen storage tanks and sufficient thermal stability to be applied at the range of temperatures necessary for gas storage applications.

## Introduction

Despite the significant interest in hydrogen-based forms of energy in the last few decades and the increased efforts to make it a valid alternative for fossil fuels in times of increasing environmental concerns, there remain a number of challenges that have to be addressed before hydrogen will become a conventional and commonly available energy carrier [1]. These challenges include (i) the energy necessary to recover molecular hydrogen from its compounds, (ii) concerns regarding safety since gaseous hydrogen is extremely flammable and will combust if its concentration in air exceeds 4% [2] and (iii) the demanding infrastructure to deliver hydrogen to consumers, which restricts hydrogen-fuelled cars from becoming a competitive alternative to conventional vehicles running on hydrocarbon fuels.

However, one of the largest obstacles is a lack of efficient, safe and convenient method to store hydrogen. Hydrogen is the lightest element in the periodic table and whilst it has a high energy density per unit mass (with a higher heating value of 142 MJ/kg), it has a low energy density per unit volume and must therefore be highly compressed or liquefied [2]. The present technology allows the maximum volumetric energy density of compressed hydrogen to achieve only 4.4 MJ/l inside the state-of-the-art Type IV pressurised tank, or liquid hydrogen to achieve 8.4 MJ/l in a cryogenic tank; this is relatively low when compared to 31.6 MJ/l for gasoline [1]. Moreover, storing hydrogen in high-pressure tanks or in liquid form in cryogenic tanks raises safety issues and requires significant materials and energy investment costs. Pressurised tanks are expensive due to their need to withstand high stresses and they occupy large volumes, which is a major drawback considering their potential application in compact vehicles. Liquid hydrogen is more space efficient but liquefaction requires low temperatures (20 K), and achieving this consumes 30% of the energy contained in the hydrogen fuel [3]. Therefore, the search for an alternative hydrogen storage method has intensified in the last decade. A variety of materials have been investigated in terms of storing hydrogen by physisorption [4, 5] with their ability to provide complete reversibility and release of hydrogen on demand being crucial for hydrogen applications, such as in the automotive sector. A number of researchers have focused on high-surface area nanoporous materials where the stored hydrogen is at high density but easily accessible; examples of such materials include zeolites [6], activated carbons [7] and metal–organic frameworks (MOFs) [8]. In this paper, we focus on porous polymeric materials which have an advantage of low intrinsic density, since they consist of only light elements. The materials also offer the possibility of efficient adsorption of hydrogen, for example, up to 2.7% H_2_ by mass at a pressure of 1 MPa and temperature of 77 K in a pure form [9]. Such uptake characteristics are comparable to those of some MOFs [10].

**Figure 1:**
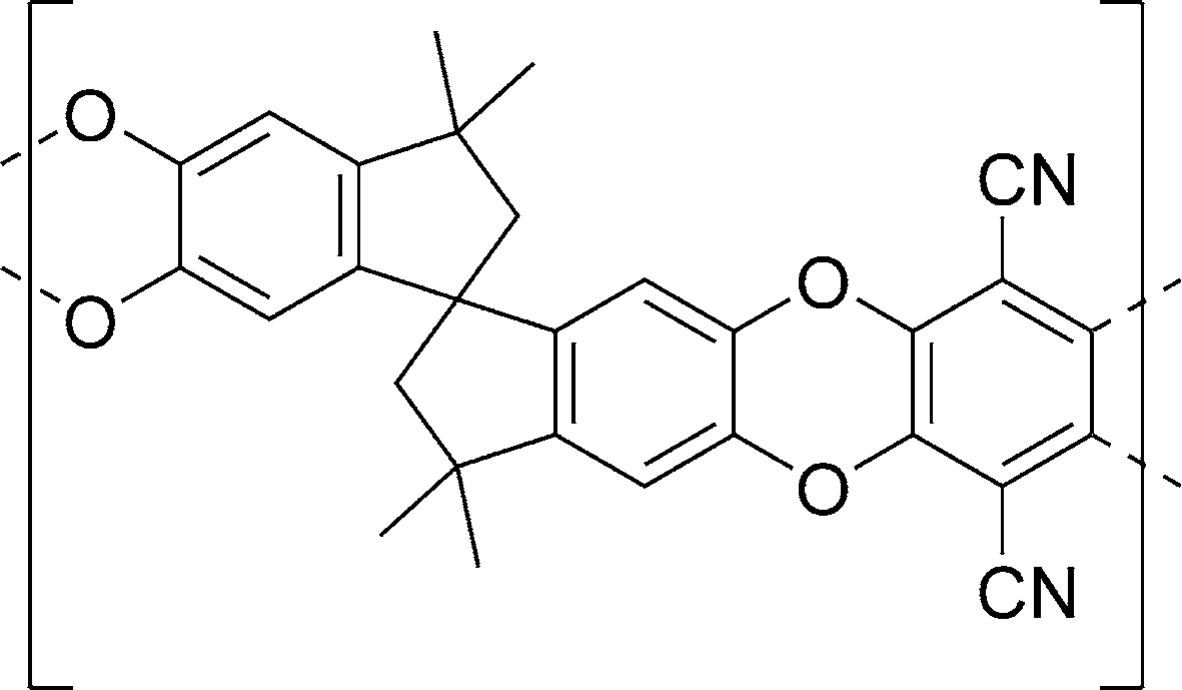
Structure of PIM-1

One class of porous polymer materials is the polymers of intrinsic microporosity (PIMs) first developed by McKeown [11]. These are classified as microporous materials (according to IUPAC, micropores are less than 2 nm in width [12]) and have been engineered in such a way that they do not display rotational freedom along the polymer backbone. The presence of a rigid but kinked structure does not allow the polymer chains to achieve efficient space packing, thereby creating pores between the polymer chains (Fig. 1). Due to the presence of the microporosity within these polymers, they exhibit high gas permeability properties. In this study, we have focussed on PIM-1 (Fig. 1), a soluble PIM that possesses one of the best surface areas in the sub-class and is synthesised from commercially available starting materials. It has been selected due to its ease of preparation and handling (solution-processability), an accessible internal surface area of approximately 800 m^2^/g and hydrogen adsorption behaviour which is rapid and reversible [10]. The properties of the material have been characterised as both an adsorbent and as a membrane since PIM-1 is a highly popular choice as a mixed matrix membrane material for gas separation membranes [13–15]. However, little attention has been paid to its mechanical properties.

Our interest in this material is based on combining a state-of-the-art high-pressure hydrogen storage tank (Type IV) with this new class of porous polymer that exhibits promising adsorption characteristics; this would be achieved by incorporating PIM-1 as a tank liner. Our aim is to understand whether such a tank liner would be advantageous in terms of tank design by either increasing the volumetric density of the hydrogen stored in the tank or decreasing the internal pressure, to enable safer and more cost-efficient compressed hydrogen storage. To achieve this, there is a need to develop a material with sufficient hydrogen uptake and mechanical properties that enable it to survive the mechanical strains when used as a tank liner. A Type IV hydrogen storage tank is designed to store hydrogen pressurised to 70 MPa and consists of a carbon composite shell providing excellent mechanical properties, and an internal high-density polymer liner which works as a gas diffusion barrier [16]. ISO safety regulations allow for range of temperatures between −40 and 85 °C and maximum filling pressures not exceeding 1.25 times the working pressure inside the tank [17]. Any material incorporated as a liner must therefore be able to withstand the internal pressure (although it may be decreased due to the adsorption phenomenon) and have sufficient extensibility to be capable of withstanding the mechanical strains of the tank that occur during hydrogen loading. The mechanical properties of the liner material must also be stable within the working range of temperatures. In addition to understanding the potential of the material as a tank liner, a mechanical characterisation of PIMs is also of interest in optimisation of the material for other applications, such as gas separation membranes.

Little work has been reported in the literature concerning the mechanical properties of polymers of intrinsic microporosity, such as PIM-1. Budd et al. performed dynamic mechanical thermal analysis (DMTA) on a PIM-1 membrane with a thickness of 40 μm obtained from 2.2 wt% solution of the polymer in tetrahydrofuran (THF) [18]. The tensile storage modulus, *E′*, was found to be approximately 1 GPa which is in the range of the moduli obtained for a glassy polymer. The storage modulus decreased slightly with increasing temperature to 350 °C, at which point the polymer degrades.

Song et al. performed tensile testing of PIM-1 films cast from a 2 wt% solution of the polymer in chloroform [19]. The tensile strength measured for an 80-μm-thick membrane was 45 MPa, and the failure strain exceeded 10%. There was no discussion on the elastic modulus but from the reported stress–strain curve it was approximately 1.2 GPa [19]. In 2010, Du et al. using the same methodology measured the tensile strength of PIM-1 films cut into dumbbell-shaped samples with a thickness 70–90 μm, obtaining a tensile strength at break of 47.1 MPa and a tensile strain at break of 11.2% [20]. No studies indicate the amount of tested samples, nor the standard deviations of properties, which are of interest to understand the variability of properties. Additionally, in the work of Song et al. [21], nanoindentation of PIM-1 films was performed resulting in a hardness of 149 ± 4 MPa and a Young’s modulus 1.876 ± 0.029 GPa. It is worth noting that the molar masses of PIM-1 used in these studies [18–21] had a range of values, as presented in Table 1.

**Table 1:** Comparison of number-average molar mass *M*_n_, weight-average molar mass *M*_w_ and polydispersity (PDI) in the reported literature

Source	*M*_n_ (g/mol)	*M*_w_ (g/mol)	PDI
Budd [18]	96,000	270,000	2.8
Song [19]	30,000	57,000	1.9
Du [20]	55,000	85,000	1.6
Song [21]	80,000–100,000	160,000–200,000	2.0
This study	76,261	193,074	2.5

In addition, whilst the porosity of PIM-1 has been well studied through the use of nitrogen adsorption isotherms, there is relatively little information reported on the ability of PIM-1 to act as a hydrogen storage material. The leading study on this matter is that of McKeown et al. [9], who, building on previous work on this subject [10, 22], demonstrated that the adsorptive hydrogen uptake of PIM-1 at 77 K to be 1.45 wt% at 10 bar. The desorption curve is described by Budd et al. [22] as showing no significant hysteresis, although no desorption curve is presented with this data.

Since there are currently only limited data on the mechanical properties of PIM materials, the aim of this paper is to perform a detailed analysis of the tensile mechanical properties of PIM-1 films in static and dynamic modes with uniaxial tensile testing and dynamic thermal analysis, respectively. To complement the material properties evaluation, a comprehensive gas adsorption study of PIM-1, in both powder and film morphologies, was performed with both nitrogen and hydrogen at 77 K in pressures up to 17 MPa.

## Materials and methods

### Polymer preparation

The method of Budd et al. [18] was used for the synthesis of PIM-1. 5,5′,6,6′-tetrahydroxy-3,3,3′,3′-tetramethyl-1,1′-spirobisindane (5.11 g, 15 mmol, 1 eq.), tetrafluoroterephthalonitrile (3.00 g, 15 mmol, 1 eq.) and anhydrous K_2_CO_3_ (16.59 g, 120 mmol, 8 eq.) were added to a two-neck 250-ml round-bottom flask fitted with a condenser. The solids were evacuated and backfilled with nitrogen three times, after which anhydrous DMF (100 ml) was added. After a few minutes a yellow precipitate started to form. The reaction was vigorously stirred for three days at 65 °C under N_2_. After cooling the reaction to room temperature, the contents were added to water (300 ml) and stirred for 1 h. The solution was filtered and the solid air-dried. Purification of the yellow PIM-1 solid was achieved via reprecipitation. The crude PIM-1 was dissolved in the minimum amount of chloroform (CHCl_3_) and re-precipitated by addition of MeOH (800 ml). This was carried out three times. The product was collected and dried in vacuo at 80 °C overnight. The molar mass of the produced polymer was determined by gel permeation chromatography against polystyrene standards on a polymer laboratories PL-GPC 50 integrated GPC system, using THF (1 ml/min at 35 °C) as the solvent.

### Film casting and samples preparation

For the evaluation of the mechanical properties of PIM-1, thin films were prepared for uniaxial tensile testing and dynamic mechanical thermal analysis (DMTA). Powdered PIM-1 does not allow processing by melting but it is soluble in CHCl_3_ and THF [11]; therefore, one of the available methods of solid polymer preparation for mechanical parameters investigation was solvent casting. PIM-1 was dissolved in CHCl_3_ in a PIM-1-to-solvent ratio of 1:50 by mass and stirred for 2 h at an elevated temperature of 60 °C until a homogenous solution was obtained. The liquid was subsequently poured into a large petri dish (200 mm diameter) and left for the solvent to evaporate. Depending on the required thickness of the resultant thin-film membrane, evaporation and desiccation of the film took up to 48 h. In order to evaluate the optimal thickness for tensile testing, polymer films were prepared with different amounts of solution with resulting thicknesses in the range of 12–65 μm. After evaporation was complete, the film had to be removed from the glass substrate. Film thickness measurements were performed multiple times along each film using an absolute Mitutoyo micrometer screw gauge with a measurement force adjustment. Samples for tensile testing were prepared according to the standard for tensile testing of polymer thin films (ISO 527-3) [23], with reduced size according to the standard on test specimens for plastics (ISO 20753:2008) [24] for reduced-scale test specimens, and were therefore in the shape of a dog-bone sample that was 75 mm long and 5 mm wide along the working region of the tensile test sample. We have also performed a comparative experiment to ensure that results obtained with small samples are comparable with those for a Type 2 specimen preferred in ISO 527-3 [23]. We obtained results of ultimate stress, strain and elastic modulus within the uncertainty limit from both types of specimens; therefore, we believe that the mechanical data obtained within this study can be compared with other studies performed according to the standard. Samples were cut from the polymer film with a scalpel along a standardised cutting form. In order to perform DMTA, samples had to be smaller and were 5 mm in width and approximately 20 mm in length, with a clamping gauge length of 5 mm. All samples were prepared from the same batch of polymer solution in order to ensure reproducibility of results.

Imaging of the evaporation of solution was undertaken in an effort to understand the morphology of the final as-cast films. This was achieved using a Leica M205 C stereo microscope to observe the surface structure and a Leica DM ILM inverted microscope to analyse the structure from the bottom during evaporation of the chloroform.

PIM-1 films for adsorptive analysis were prepared by dissolving 0.2 g of PIM-1 powder in 5 ml of CHCl_3_, and stirring at room temperature until completely dissolved. The film was left for 48 h to cast, before being cured at 70 °C for 24 h, in order to remove any remaining solvent.

### Gas adsorption study

Brunauer, Emmett and Teller (BET) surface areas and pore size distributions (PSDs) were determined from nitrogen (N_2_) isotherms at 77 K, which were performed on a Micrometrics ASAP 2020 volumetric gas sorption analyser. The samples were degassed under high vacuum at 180 °C for 8 h in order to remove any adsorbate and residual solvent within the material. BET surface areas were determined from the data using the British Standard BS IS0 9277:2010 [25], although the range of *P/P*_0_ (where *P* is the absolute pressure and *P*_0_ the vapour pressure of the adsorptive at the isotherm temperature) chosen was determined using the consistency criteria provided by Rouquerol et al. [26] to better reflect the expected microporous nature of the material. Both the surface area and PSD were calculated in Micromeritics MicroActive 1.01 software.

High-pressure hydrogen isotherms (up to 17 MPa) were analysed using a Hiden Isochema HTP-1 Sievert’s-type volumetric sorption measurement system. All isotherms were performed at 77 K, using immersion in a liquid nitrogen dewar as the temperature control mechanism. The samples were prepared by loading the sample chamber with either powder or film (cut into ~20 mm × 5 mm pieces), and degassed under the same conditions as described above. All hydrogen uptakes are reported as sample-specific excess uptakes, i.e. relative to the dry sample mass.

### Mechanical testing

Static uniaxial tensile tests were performed using an Instron 3369 tensile testing machine with a 100 N static load cell (62,291). Prior to mechanical testing, the dimensions of all samples were measured using the micrometer screw gauge. The tensile test was performed for all specimens with a quasi-static speed of 2 mm/min at an ambient temperature of 20 °C. Samples exhibiting any structural damage, notches or slipping in clamps, were excluded from the results. To gain an understanding of the variability of the mechanical properties, 20 samples were tested.

DMTA was performed using a Mettler Toledo DMA1 Star System with a liquid nitrogen cooling functionality. Experiments were performed in a tension mode with an oscillatory displacement of 25 μm (strain 0.005) applied to a sample with a frequency of 1 Hz. In addition, an upper limit of tensile force was set to 10 N so that the displacement was smaller when this force limit was exceeded. Samples were cooled to a temperature of −150 °C with liquid nitrogen, and the dynamic mechanical response of the sample was recorded at a ramp rate of 5 °C/min, up to the decomposition temperature of the material. The experiment was repeated six times on the samples cut from the same sheet of PIM-1 film, in the same direction, to minimise the influence of external factors or film anisotropy on the experiment results.

### Scanning electron microscopy imaging

Failure surfaces of samples ruptured during the tensile testing were analysed using scanning electron microscope (SEM) JEOL JSM6480LV in order to investigate the damaged edge surface and acquire more information on the deformation and fracture process, and the internal structure of the PIM-1 thin-film membranes. As a control case to examine the internal structure, we performed additional imaging on samples that were bisected with tearing. Samples were gold coated to avoid sample charging in the scanning electron microscope.

## Results and discussion

### PIM-1 polymer characterisation

For the PIM-1 material synthesised in this study, the number-average molar mass (*M*_n_) was determined to be 76,261 g/mol, and the weight-average molar mass (*M*_w_) was 193,074 g/mol, giving a polydispersity index (PDI, *M*_n_/*M*_w_ *)* of 2.5. By comparing (Table 1) the weight-average molar masses for PIM-1 obtained in other studies, Budd et al. reported *M*_w_ = 270,000 g/mol [18], and PIM-1 mechanically tested by the group of Song and Du had *M*_w_ = 57,000 g/mol [19] and *M*_w_ = 85,000 g/mol [20], respectively, indicating we had successfully synthesised PIM-1 suitable for film formation and characterisation.

### Film casting and thickness measurement

**Figure 2:**
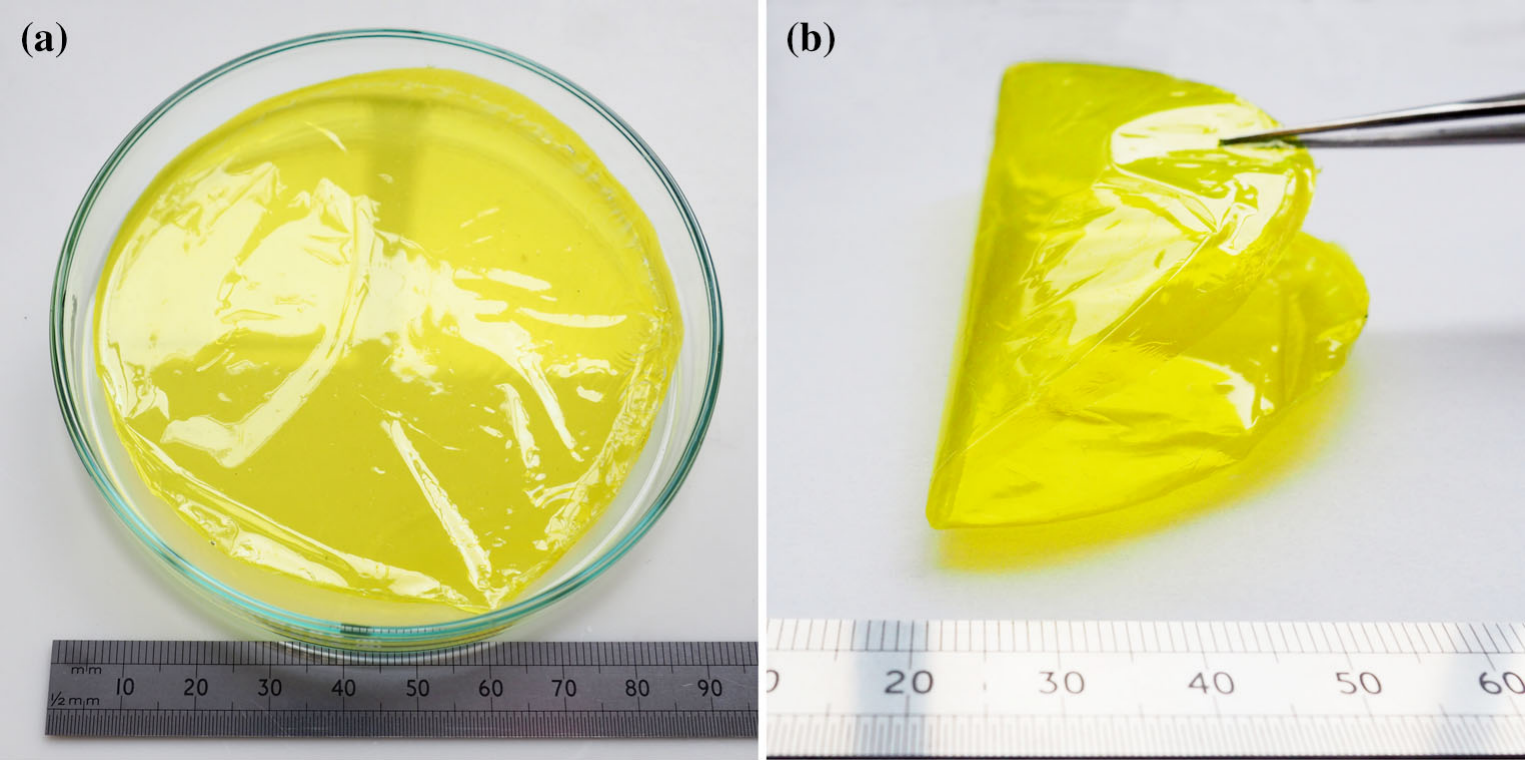
**a** Image of PIM-1 film in a small petri dish (120 mm diameter), **b** folded film showing flexibility with no failure (thickness 37 μm)

**Figure 3:**
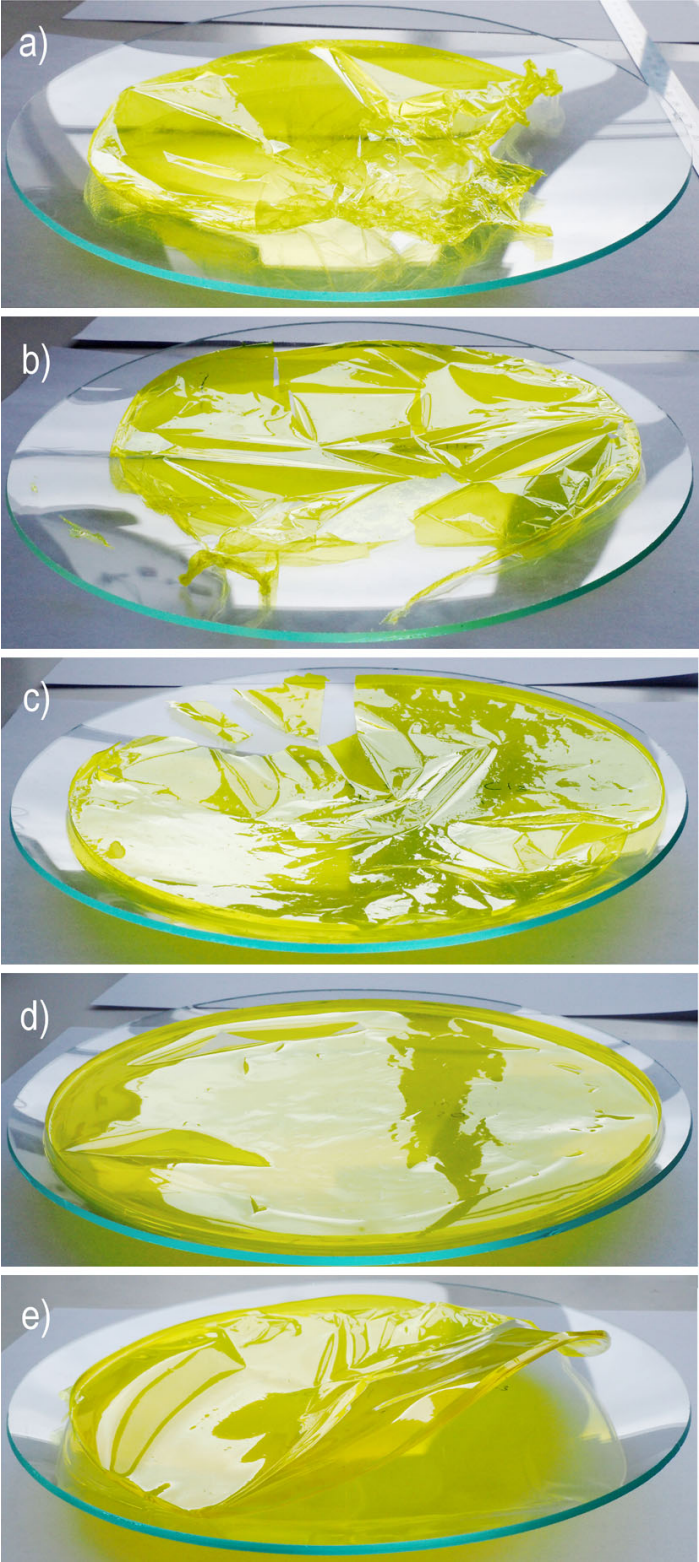
Films prepared with different amounts of solution forming membranes which have the following thicknesses: **a** 12.03 μm (too thin), **b** 18.43 μm (too thin), **c** 22.80 μm (optimal), **d** 41.97 μm (optimal) and **e** 65.07 μm (too thick). The diameter of films was around 200 mm for scale

After casting and evaporation, the PIM-1 film was transparent and had a bright yellow colour as shown in Fig. 2. These films could be deformed to a high curvature (Fig. 2a). It was found that films with a thickness over 60 μm were liable to shrinkage and corrugation during the evaporation process, and this prevented the formation of flat samples, free from stress concentrations which would influence the tensile testing data. However, when the film thickness was below 20 μm, it had insufficient mechanical strength and was easily damaged during removal from the glass petri dish and handling. Therefore, it was determined that optimisation of the film thickness parameter was crucial for the formation of planar and defect-free thin films to form appropriate mechanical test specimens. Five films with varying solution volumes were produced and measured in terms of their thickness as shown in Table 2. It was found that the optimal thickness for obtaining good quality films with sufficient mechanical strength for sample preparation and testing was between 25 and 45 μm. The films prepared with different amounts of solution resulting with different thicknesses are shown in Fig. 3. It can be clearly observed that three thinnest films (12, 18 and 22.8 μm in thickness) followed the shape of the watch glass and deformed under their own weight. The 12-μm film was very fragile, and could not be straightened due to damage caused by handling. The film with a thickness of approximately 40 μm maintained horizontal alignment, despite the watch glass shape, due to increased stiffness. The thickest sample, with average thickness of 65 μm, was distorted and deformed upwards on evaporation of the chloroform.

**Table 2:** Thickness measurements of films obtained from 2 wt% solution of PIM-1 in chloroform, with their standard deviations and comparison to literature sources

	This work 10 ml	This work 20 ml	This work 30 ml	This work 40 ml	This work 50 ml	Budd [18]	Song [19]	Du [20]
Average thickness (μm)	12.03	18.43	22.80	41.97	65.07	50	80	70–90
Standard deviation (μm)	3.81	5.79	6.22	12.28	16.24	–	–	–

### PIM-1 adsorption characterisation

**Figure 4:**
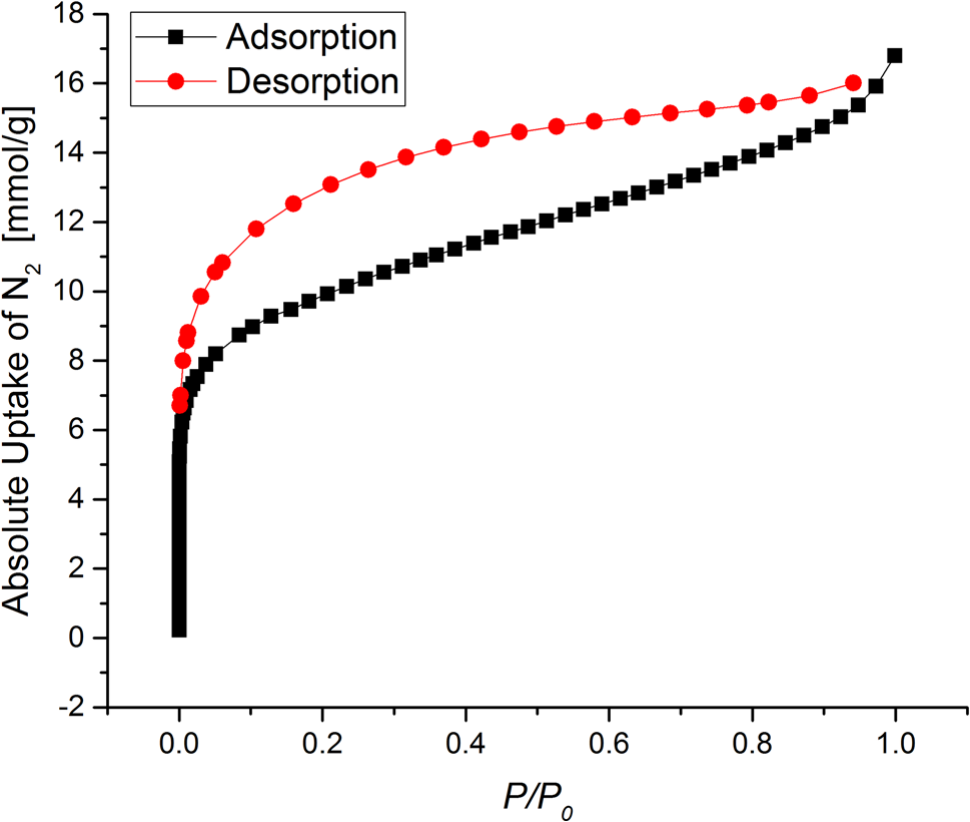
Nitrogen isotherm of PIM-1 powder at 77 K. *Lines* are provided to guide the eye

The nitrogen isotherm for a powder sample of PIM-1 is shown in Fig. 4. This isotherm shares many qualities with those previously reported, namely the combination of IUPAC Type I and Type IVa behaviour, displaying strong porosity in both the microporous (pore diameter <2 nm) and mesoporous (pore diameter 2–50 nm) ranges [18, 22, 27]. This combination of behaviour is seen in the sharp rise in uptake in the low *P*/*P*_0_ range, followed by a more linear rise through the middle of the isotherm. This isotherm displays very large hysteresis in the desorption curve, which is commonly seen for PIM-1 isotherms, and is likely indicative either of ‘throats’ in the porosity (narrow channels that limit the passage of gas molecules), or pore swelling caused by the presence of the adsorbate [18, 22].

The BET surface area calculated for this material is 804.0 ± 2.4 m^2^/g, taking a *P*/*P*_0_ range of 0.01–0.14. This range was determined based on the criteria of Rouquerol et al. [26]. As a check for validity, the authors suggest calculating the relative pressure at which monolayer coverage is assumed to complete, which is calculated using the formula [28]:

$$ \left( {\frac{P}{{P_{0} }}} \right)_{{n_{\text{m}} }} = \frac{1}{1 + \sqrt C }, $$

where *n*_m_ is the monolayer capacity and *C* is the BET parameter. As the calculated value for *C* in this pressure range is 349.9, the relative pressure at monolayer coverage is calculated to be 0.0507, which is well within the range of pressures selected and so validating this choice. This BET surface area value compares well with those reported in the literature, as surface area values for PIM-1 powders are typically reported in the range of 750–860 m^2^/g [9, 18].

**Figure 5:**
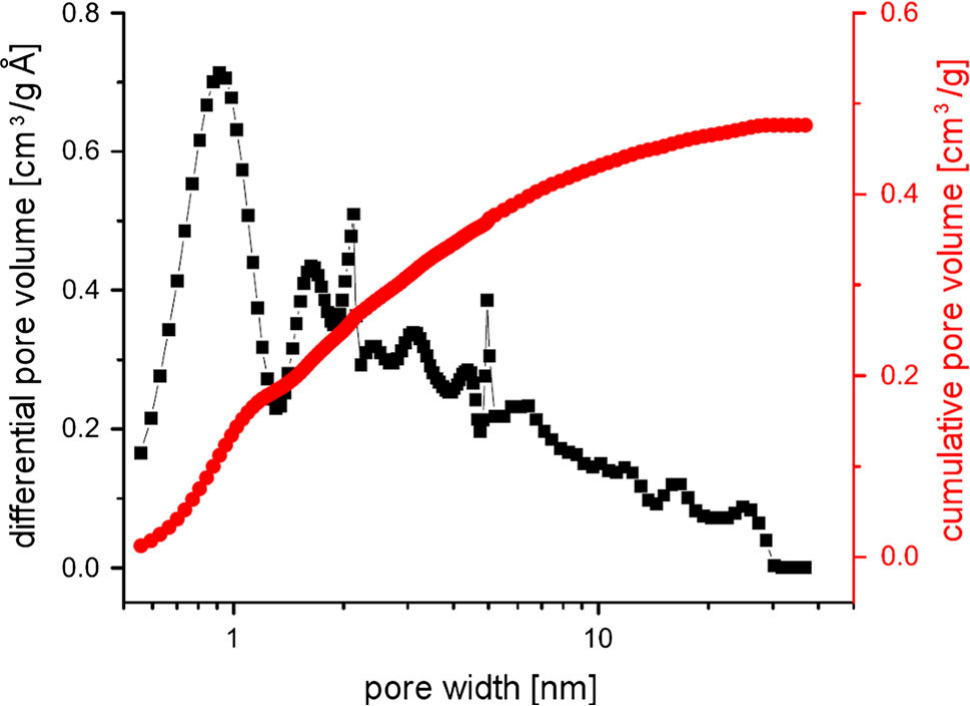
NLDFT pore size distributions calculated from the isotherm in Fig. 4. *Lines* are provided to guide the eye

The pore size distribution for PIM-1 powder, calculated using the non-linear density functional theory (NL-DFT) model provided by the MicroActive software, is shown in Fig. 5. The distribution over the nanopore range (≤100 nm) is very broad, clearly demonstrating the wide range of porosity suggested by the initial isotherm. There are significant peaks at 0.92, 1.63, 2.13 and 4.95 nm within both the microporous and mesoporous ranges. The cumulative pore size, a measure of the total available pore volume in pores up to a certain width, gives a microporous pore volume of 0.25 cm^3^/g, and a total nanopore volume of 0.43 cm^3^/g. The total pore volume as estimated by total adsorption at *P*/*P*_0_ = 0.999 is 0.583 cm^3^/g. These values compare reasonably with previously demonstrated values for both microporous and total pore volume in PIM-1 powder samples (total pore volume of Budd et al.’s sample was 0.68 cm^3^/g at *P*/*P*_0_ = 0.98, with a “significant proportion of micropores with dimensions in the range of 0.4–0.8 nm” [18]).

Full N_2_ isotherms were not successfully completed on PIM-1 film samples, as it took an impractically long time for the volumetric system to equilibrate in the low-pressure ranges. It is believed this is due to a low rate of mass transfer, due in part to the low temperatures at which the isotherm is being performed, and the distorted, tortuous nature of the porosity within the film which adds length and complexity to the diffusion pathway for the adsorptive molecules.

**Figure 6:**
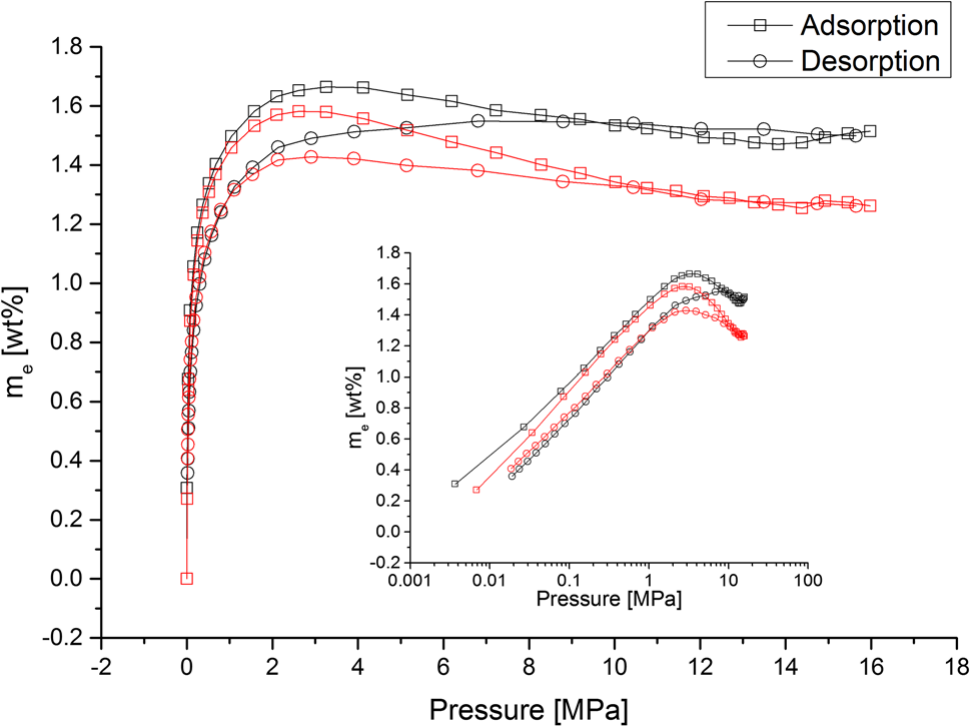
Excess hydrogen uptake on PIM-1 powder (*black*) and film (*red*) samples at 77 K. *Lines* are provided to guide the eye

The high-pressure hydrogen isotherm can be seen in Fig. 6. The adsorption curve for PIM-1 powder, seen in black, follows a very typical excess isotherm shape for supercritical adsorption, reaching 1.49 wt% at 1 MPa, and a maximum of 1.66 wt% at 3.2 MPa. Previous literature has only tested hydrogen adsorption of PIM-1 up to 2 MPa, reaching a maximum uptake of 1.45 wt% at 1 MPa [10]. The excess uptake then declines as the pressure increases due to a greater rate of density increase in the bulk phase than in the adsorbed phase [29].

The adsorption uptake in the PIM-1 thin film samples initially performs identically to the powder samples, reaching an uptake of 1.59 wt% at 2.9 MPa before the excess decreases. This lower maximum uptake and subsequent higher drop in the excess adsorbed with pressure is likely to be due to the reduced available pore volume in the films, which is caused by the full relaxation of the polymer chains during the solvent casting process, eliminating any mesoporosity formed in the powder during the reprecipitation and leaving only the intrinsic microporosity [18, 30].

In addition, the desorption of hydrogen shows an unusual trend. Whilst it also generally follows an excess curve, it is irreversible compared to the adsorption curve. Initially, the desorption curve rises above the adsorption curve in the high-pressure region, before decreasing and otherwise not showing the peak in uptake demonstrated in the adsorption curve. This desorption curve continues to be lower than the adsorption all the way through to the low pressures (<0.2 MPa).

Whilst it is unknown what causes this irreversibility, it is feasible that a change in the polymer structure is the cause, perhaps due to polymer swelling. This idea is in part suggested because the effect is reversible by degassing; repeat runs on the sample following removal of the adsorbate show identical adsorption/desorption curves. This is true for multiple attempts, with degassing performed between each run. This phenomenon may be further tested by the running of adsorption/desorption cycles without degassing in between; this work is currently in progress.

To our knowledge, very little hydrogen desorption data on PIMs is available in the literature, despite this behaviour being important in understanding how a material may behave in a hydrogen storage system. Only Budd et al. [22] have described hydrogen desorption data (in the limited pressure range up to 1.5 MPa), in which they describe the hydrogen uptakes shown as having “no significant hysteresis”. Understanding the desorption response is an important step in understanding the response of the material to the cyclic loading and unloading of hydrogen within a working storage tank.

### Static tensile tests

**Figure 7:**
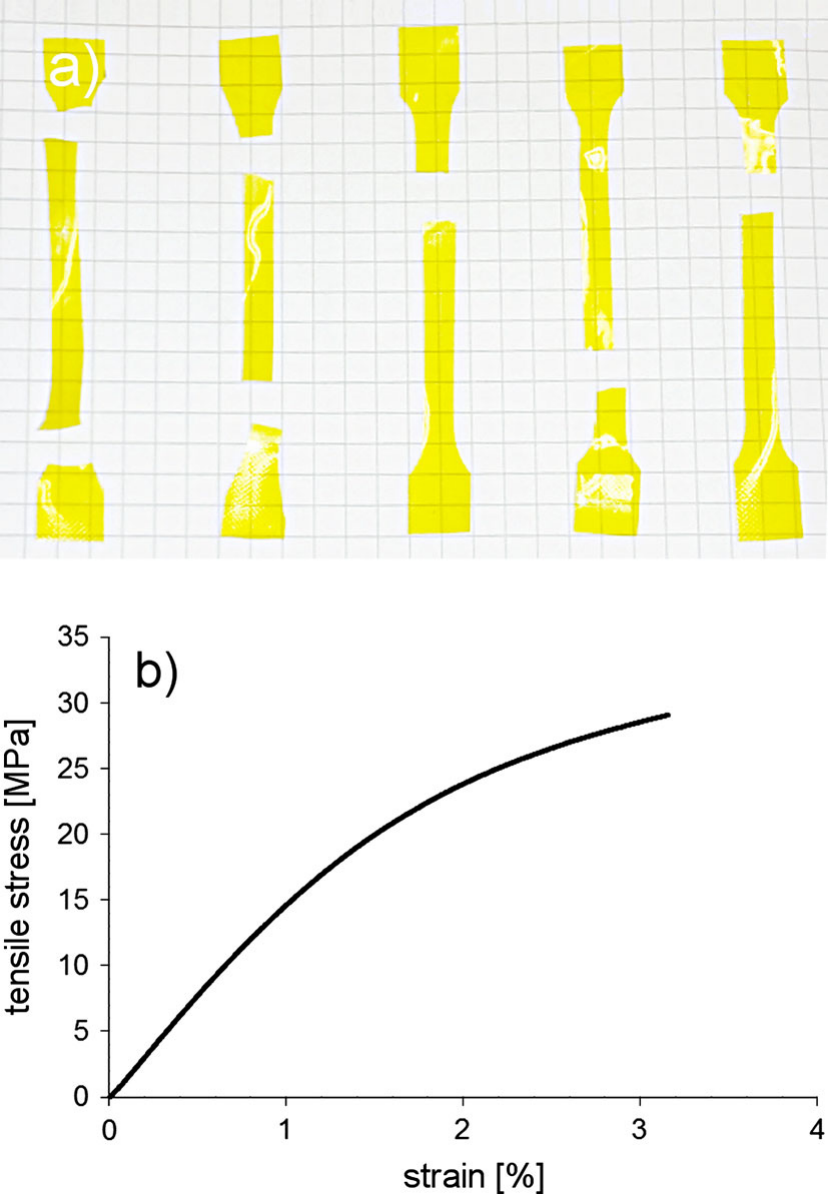
**a** Set of samples after rupture in the tensile test showing two samples from the *left* that broke simultaneously at two levels and the samples that typically broke on a single level on the *right* and **b** typical stress–strain curve obtained within the tensile test

A total of 20 PIM-1 samples were tensile tested. The measured range of sample thickness was between 43 and 143 μm with average of 76 ± 25 μm. All samples were tested until rupture and most failed in the specimen centre, whilst a small number of samples broke at two positions simultaneously (Fig. 7a). This behaviour may suggest an equal distribution of the mechanical properties along the membrane, as two distinct pieces of the sample exhibited equal strength. The average ultimate tensile stress was 30.9 ± 5.4 MPa with strains to failure reaching 4.4 ± 2.0%; the maximum observed extension at break was 3.4 mm. These values are close to the mechanical properties of bulk polystyrene, which reaches a tensile stress of 40–48 MPa and a failure strain of 6–7% [31]. However, it should be taken into account that the mechanical properties of films do vary from those measured for bulk polymers, for example, polystyrene thin films exhibit a yield strength of 88 MPa [32]. For our PIM-1 samples, the yield strength had an average of 11.0 ± 1.9 MPa and failure strains of typically 1%. The Young’s modulus obtained for all samples had uniform values, on average exceeding 1.2 GPa, with standard deviation of 130 MPa, which shows high reproducibility of results.

The values of ultimate tensile strength and failure strain obtained in this study were lower than those reported in the work of Song et al. [19] and Du et al. [20], whilst our samples exhibited a higher Young’s modulus (Table 3). The ultimate tensile strength was over 30% lower (30.9 MPa compared to 47.8 and 47.1, respectively), strain at rupture around 66% lower (4.4% compared to 10% and 11.2%, respectively), whereas the average modulus of elasticity was 27% higher when compared with Young’s modulus estimated from stress–strain curve provided by Song (1.26 GPa compared to 1.15 GPa) [19]. Both studies [19, 20] used the same solution concentration as in this work and chloroform as the solvent, but the number-average molar mass and weight-average molar mass were lower (see Table 1). We believe that the most significant effect influencing the reported differences in ultimate stress and strain values may be associated with evaporation process and its rate. Rapid solvent evaporation may cause formation of macropores and cavities in bulk structure of polymer (Figs. 9, 10) thus introducing inhomogeneities, which are reflected in decreased mechanical properties. However, macrostructural features of PIM-1 films have not been investigated in previous studies which prevents direct comparison of internal film structures.

**Table 3:** Tensile testing results of PIM-1 film compared with values reported in the literature

	Stress at break (MPa)	Strain at break (%)	Yield strength (MPa)	Yield strain (%)	Young’s modulus (GPa)	Samples tested
Average ± SD	30.9 ± 5.4	4.4 ± 2.0	11.0 ± 1.9	1.1 ± 0.3	1.26 ± 0.13	19
Range	18.2–42.2	2.1–6.5	6.6–15.6	0.4–2.0	1.07–1.58	
Song [19]	47.8	10.0	15.0^a^	1.3^a^	1.15^a^	–
Du [20]	47.1	11.2				–

^a^ Values not given by author, only estimated from the stress–strain curve

The mechanical properties measured here should satisfy the strain requirements for hydrogen storage tank liners. Taking into account a significant hydrogen adsorption of the polymer which would decrease the pressure and strains present in a 70 MPa hydrogen tank, the obtained level of flexibility and strain to failure is sufficient for a PIM-1 film to perform successfully as the tank liner. The component present in a Type IV hydrogen storage tank with the lowest strain to failure is the carbon fibres with a failure strain between 0.4 and 1.9% [33], which is significantly below the average 4.4% obtained for PIM-1 films. The lowest extreme failure strain reported in this study was not below 2.1%. However, further structural optimisation of PIM-1 has to be performed before applying it as a tank liner which will certainly influence the liner mechanical properties.

### Dynamical mechanical thermal analysis

**Figure 8:**
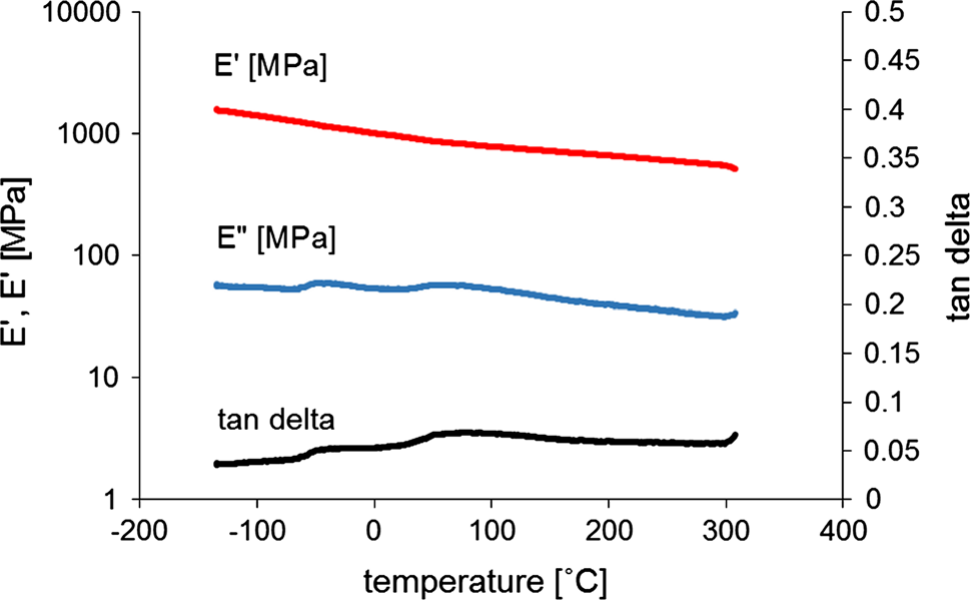
Storage modulus (E′), loss modulus (E′)′ and tan *δ* dependency on temperature (Sample 1 in Table 4)

Dynamic mechanical thermal analysis of PIM-1 thin films was performed in tension configuration. Six samples were cooled to a temperature below −150 °C using liquid nitrogen and dynamically tested with increasing temperature until eventual failure of the sample at high temperature, as exhibited by a sharp decrease in the reaction force produced by the sample as the response to a constant applied displacement. This transition occurred on average at 354 °C, when the samples were subject to significant degradation and changed from an intense yellow to a black colour and failed in a brittle manner. Until the point of sample failure at high temperature, there was no other rapid transition observed which confirmed the results obtained by Budd et al. where decomposition occurred at around 350 °C [18]. Two small peaks were observed in storage modulus curves at around −50 and 80 °C which might indicate a mild transition (i.e. glass transition). The second peak was also visible in the curve presented in [18]. However, this transition was insignificant to mechanical behaviour of the sample that might influence its performance in intended applications. Table 4 summarises the DMTA data and shows some variation of the modulus values for all samples. Two values describing the modulus are reported: the average modulus over the temperature range and the modulus measured at 20 °C (Table 4). In general, tan *δ*, which is a measure of energy dissipation in a material, was relatively low (0.05 ± 0.004) which means that stress and deformation were nearly in phase. This in turn suggests that the complex modulus E* is almost equivalent to the storage modulus *E′*, whereas the loss modulus *E*′′ is low which indicates that our samples did not manifest significant viscoelastic behaviour in the temperature range examined and can be considered almost purely elastic. The averaged complex and storage moduli was over 960 MPa with a standard deviation not exceeding 240 MPa. Even though the obtained values of storage modulus are close to those reported in the literature (1 GPa), it should be noted that another solvent (tetrahydrofuran) was used for the preparation of samples in the work of Budd et al. [18]. The influence of the solvent on polymer films’ mechanical properties has been reported for such thermoplastics as PLA [34] and polystyrene [35]; therefore, this may also be the case for PIM-1. It is also necessary to consider the influence of polymer treatment on the results, as polymers are susceptible to processing history and every step, from polymer preparation in granular form to diluting it in different solvents, casting and drying in different conditions, may be significant to a final outcome. However, the shape of the curves and the tendency of the moduli to decrease with increasing temperature are consistent with those presented previously [18]. A typical example of such a DMTA curve is shown in Fig. 8.

**Table 4:** Results of dynamic mechanical thermal analysis of PIM-1 film obtained in this study and in the study of Budd et al. [18]

Sample	Thickness (μm)	Max T (°C)		E′ (MPa)	E″ (MPa)	E* (MPa)	tan *δ*
A1	44 ± 5	385	20 °C	738	38.8	739	0.052
			Av	664	38.0	665	0.057
A2	42 ± 4	350	20 °C	1363	51.1	1364	0.037
			Av	1249	54.2	1250	0.049
B1	54 ± 4	355	20 °C	975	30.0	976	0.031
			Av	892	35.4	893	0.044
B2	44 ± 5	337	20 °C	884	41.4	885	0.047
			Av	843	42.1	844	0.050
C1	62	348	20 °C	672	30.4	673	0.045
			Av	619	32.0	620	0.051
C2	63	348	20 °C	1172	34.1	1172	0.029
			Av	1057	46.0	1058	0.047
Average	51.5	353.8	20 °C	967.2	37.63	968.0	0.0403
			Av	887.2	41.28	888.3	0.0498
St dev	8.7	15.0	20 °C	240.0	7.31	239.9	0.0086
			Av	217.3	7.30	217.5	0.0041
Budd [18]	40	350		1000			

Moduli and tan *δ* values given at 20 °C and as average (Av) in the whole temperature range. Average value of storage modulus is 887.2 MPa over wide range of temperature whereas storage modulus obtained at 20 °C is 967.2 MPa

### Microscopy analysis of PIM-1 samples

**Figure 9:**
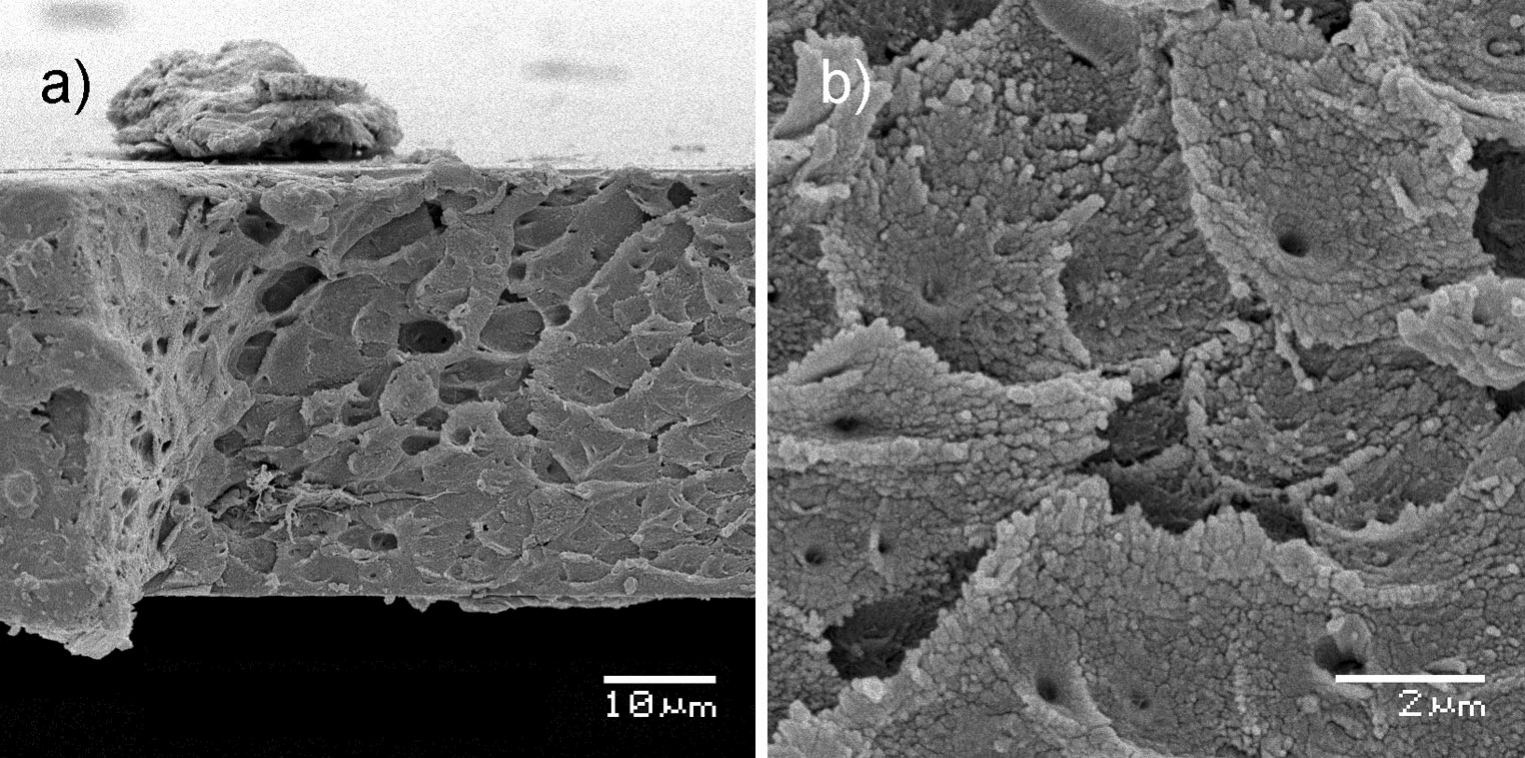
SEM of the sample fracture surface, **a** low magnification, **b** high magnification

The intrinsic microporosity of PIM-1 has been measured in terms of accessible surface area and gas adsorption but micropore sizes (less than 2 nm [12]) are below the resolution of scanning electron microscopy (SEM). Examination of the failure surfaces of PIM-1 after being subjected to tensile testing has shown high roughness and a layered structure with distinct ‘bumps’, and the ruptured surface clearly distinguishes itself from the defocused upper surface visible in the background (Fig. 9a) which was flat and uniform. At higher magnification, cavity structures with small round holes in the middle of the features were observed which appeared to be a cone-like structure (Fig. 9b). The roughness of the surface was similar along the edge, sometimes with larger bumps and level changes as shown in Fig. 9a.

**Figure 10:**
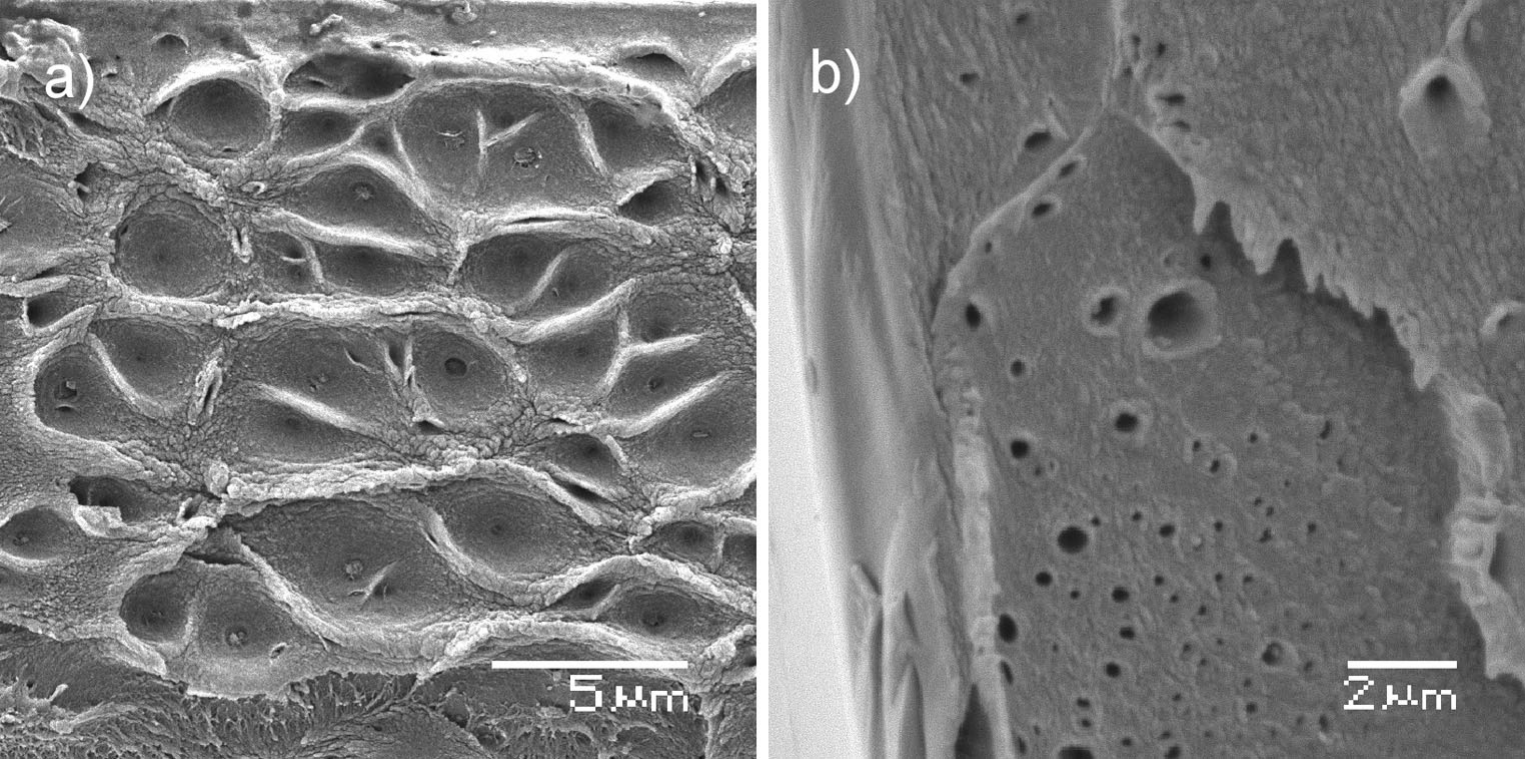
Scanning electron images of PIM-1 film sample cross-section showing internal mesoscopic porosity obtained with cutting (**a**) and tearing (**b**)

In order to see if SEM observations of the failure surface of the tensile test specimen are tensile damage specific and related to the failure mechanism, we prepared control samples, where cross-sections were obtained by cutting and tearing the sample. One feature of the surface that was clearly observed in all samples was the presence of holes with diameters in range of 0.1–1 μm. All samples exhibited a porosity in the macro scale along the cross-section surfaces; however, these macropores are too large to introduce effective additional surface area in the presence of intrinsic micropores. Nevertheless, they may be an efficient access route for hydrogen to the micropores at the nanoscale, allowing the hydrogen molecules to penetrate inwards the film and improve the hydrogen adsorption. The distribution of the macropores was not highly regular, due to rather chaotic nature of the evaporation process, but the pores were evident along the cross-section. It was more common for the pores to appear in the centre part of the film, rather than at the film edges (Fig. 10).

**Figure 11:**
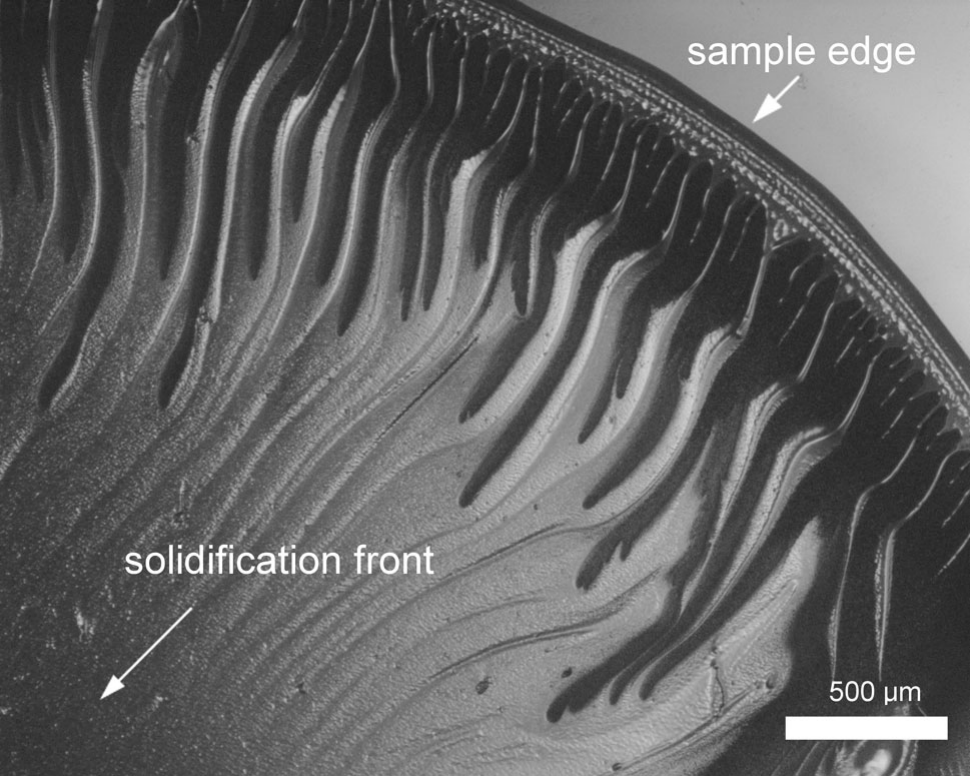
Solution of PIM-1 in chloroform during evaporation observed with a stereo microscope

We also observed that the film surface is homogenous and does not exhibit the macroporosity in contrast to the cross-sectional surface (Fig. 9a, top of the picture) of the tensile test specimens. This may suggest that there exists a denser, less porous outer layer formed during the relaxation of polymer chains during solvent evaporation that hinders gas adsorption process and this may explain the lower adsorption rates of PIM-1 films when compared to PIM-1 powder (1.59 wt% vs. 1.66 wt%). The observed cavities may result from the evaporation process during film formation. Evaporation of chloroform is relatively rapid and leads to the formation of secondary structures at the macro-scale level. In order to examine this hypothesis, we performed microscopic imaging of an evaporating solution in real time. When a drop of the PIM-1 and chloroform solution was observed under a transmission optical microscope, the polymer solidification was seen to clearly progress from the sample edges towards the centre. During the process, it could be observed that a rough/corrugated structure was formed on the surface of the dry PIM-1 resembling wrinkles and creases (Fig. 11). Due to a limited possible depth of the imaged sample, we performed the experiments with a small amount of solution. This mechanism of progressing, rapid solidification could be also observed at the macro scale when forming large surface films; therefore, the creased characteristics might be extrapolated to the larger scale sample case. This behaviour most possibly explains the appearance of holes and cavities inside the film observed with SEM imaging. The presented structural analysis might be useful in determining the optimal conditions to prepare films with possibly highest surface-to-volume ratio by adjusting solution concentration and evaporation surface.

## Conclusion

This study has demonstrated the successful synthesis and characterisation of PIM-1 as a base material for use in a hydrogen storage system. The polymer has been cast into films with ease, and N_2_ adsorption experiments have demonstrated that the porosity of the produced material is comparable to that of PIM-1 samples reported by other groups. High-pressure hydrogen adsorption analysis has shown an interesting adsorption/desorption behaviour, with uptakes comparable to those previously reported. However, this study has demonstrated hydrogen adsorption at −196 °C up to pressures not previously reported for this material (to 17 MPa), and therefore demonstrates the maximum excess adsorbed capacity of the material, and its uptake behaviour in higher pressure regimes. The desorption behaviour has also demonstrated a highly unusual characteristic curve, the reasons for which are yet to be determined.

We have undertaken the first thorough mechanical characterisation of PIM-1 film using standard methods for materials testing such as uniaxial tensile testing, dynamic mechanical thermal analysis (DMTA) and imaging of films’ mesoporous structure and fractography. The obtained results show the potential for high reproducibility, especially within tensile testing strength and Young’s modulus data where standard deviations are typically 17 and 10%, respectively. Observed differences obtained in mechanical properties compared to other studies may be attributed to the structural properties of cast films and the presence of macropores which were observed in this study which are likely to influence the mechanical behaviour such as tensile strength and strain to failure. Other factors influencing the results are polymer treatment history, solvent used for film casting and the evaporation process within which the film is being formed. Scanning electron microscopy analysis revealed interesting structural patterns with secondary mesoscale porosity which might increase the capabilities of material to adsorb hydrogen by providing additional access routes, but further investigation has to be conducted in order to determine if and how this mesoporosity can be regulated.

Results suggest that PIM-1 has sufficient elasticity to withstand deformations occurring within state-of-the-art high-pressure hydrogen storage tanks and good thermal stability to be applied over a wide range of temperatures which is necessary for mentioned application. With further improvements in gas adsorption capacity, for example, using PIM-1 as a matrix in a formable, elastic composite containing high surface area, particulate fillers such as a metal organic framework (MOF) or covalent organic framework (COF), and structural optimisation, there are strong indicators that this material will be a basis of future developments in hydrogen storage in high-pressure tanks.
